# Supplementary material for: Leveraging nonlinear relationships and interactions to improve 30-day pneumonia readmission machine learning models
Source: PLoS One. 2026 Jun 5;21(6):e0349804. doi: 10.1371/journal.pone.0349804 (PMC13240904; doi:10.1371/journal.pone.0349804)
Supplement: S1 Table — This table contains all study variables considered for use as model features. The left column is the category (e.g., demographics). The right column is the individual variable (e.g., patient sex). (DOCX) [file pone.0349804.s001.docx]

**S1 Table. Study variables**

| **Category** | **Description** |
| --- | --- |
| Demographics | Age in years at index visit discharge |
|  | Patient sex |
|  | Insurance type  Medicaid, Medicare, other |
|  | Spoken language  English, Spanish, other |
|  | Marital status  married, divorced, single, other |
|  | Employment status  not employed, employed, retired, disabled, other |
|  | Body mass index (kg/m^2^) at index visit |
| Encounter information | Index visit length of stay |
|  | Pre-index visit utilization frequency: emergency |
|  | Pre-index visit utilization frequency: inpatient |
|  | Pre-index visit utilization frequency: ICU |
|  | Pre-index visit utilization frequency: observation |
|  | Pre-index visit utilization frequency: mental health |
|  | Pre-index visit utilization frequency: primary care |
|  | Pre-index visit utilization frequency: specialty |
|  | Pre-index visit utilization frequency: any outpatient |
|  | Pre-index visit utilization frequency: other |
| Comorbidities | Acute kidney failure |
|  | AIDS/HIV |
|  | Alcohol abuse |
|  | Blood loss anemia |
|  | Cancer, no metastasis |
|  | Cancer, metastasis |
|  | Cardiac arrhythmias |
|  | Cerebrovascular disease |
|  | Chronic pulmonary disease |
|  | Coagulopathy |
|  | Congestive heart failure |
|  | Deficiency anemia |
|  | Dementia |
|  | Depression |
|  | Diabetes, complicated |
|  | Diabetes, uncomplicated |
|  | Drug abuse |
|  | Fluid and electrolyte disorders |
|  | Hypertension, complicated |
|  | Hypertension, uncomplicated |
|  | Hypothyroidism |
|  | Liver disease, mild |
|  | Liver disease, moderate or severe |
|  | Lymphoma |
|  | Myocardial infarction |
|  | Nicotine dependence, current |
|  | Nicotine dependence, history |
|  | Obesity |
|  | Other neurological disorders |
|  | Paralysis |
|  | Peptic ulcer disease |
|  | Peripheral vascular disorders |
|  | Psychoses |
|  | Pulmonary circulation disorders |
|  | Renal disease |
|  | Renal failure |
|  | Rheumatoid arthritis/collaged vascular disease |
|  | Rheumatoid disease |
|  | Sickle cell disease |
|  | Valvular disease |
|  | Weight loss |
| Medications | Long term (current) use of insulin |
|  | Long term (current) use of anticoagulants |
|  | Long term (current) use of antithrombotics/antiplatelets |
|  | Long term (current) use of other drug therapy |
| Vital signs, first and last | Temperature (⁰F) |
|  | Systolic blood pressure (mm Hg) |
|  | Diastolic blood pressure (mm Hg) |
|  | Respiration (breaths/min) |
|  | Pulse (beats/min) |
| Laboratory results, first and last | Creatine (mg/dL) |
|  | BUN (mg/dL) |
|  | Hemoglobin (g/dL) |
|  | Hemoglobin A1C (mg/dL) |
|  | Hematocrit (L/L) |
|  | MCHC (g/dL) |
|  | Sodium (mmol/L) |
|  | White cell count (10^9 cells/liter) |
| Geographic information | Hispanic origin, % |
|  | Non-Hispanic White, % |
|  | Non-Hispanic Black, % |
|  | Foreign born, % |
|  | ≥5 years old who don't speak English well, % |
|  | Less than high school diploma, % |
|  | High school diploma and/or some college, % |
|  | Bachelor’s degree or higher, % |
|  | Families with income <$40,000, % |
|  | Families with income $40,000 to $74,999, % |
|  | Families with income $75,000 to $124,999, % |
|  | Families with income $125,000 or more, % |
|  | ≥15 years old and never married, % |
|  | <18 years old, % |
|  | ≥70 years old, % |
|  | ≥16 years old and unemployed, % |
|  | Income in the past 12 months below poverty level, % |
|  | Households with public assistance income or food stamps, % |
|  | Female-headed families with children, % |
|  | Single parent families with children, % |
|  | Owner occupied housing units, % |
