# Supplementary material for: Leveraging nonlinear relationships and interactions to improve 30-day pneumonia readmission machine learning models
Source: PLoS One. 2026 Jun 5;21(6):e0349804. doi: 10.1371/journal.pone.0349804 (PMC13240904; doi:10.1371/journal.pone.0349804)
Supplement: S2 File — This file contains detailed information regarding the development of XGBoost and deep neural network models. Specifically, the training procedure and hyperparameter ranges utilized for development are provided. (DOCX) [file pone.0349804.s002.docx]

**S2 File. Model hyperparameter tuning details**

For all models, we executed the identical marginal screening procedure and cross validation procedure (i.e., five folds, optimization for AUROC performance).

**XGBoost [1]**. We tuned the following hyperparameters using an exhaustive grid search. We varied learning rate between 0.001, 0.01 and 0.10. We varied tree depth between 2-12 by 2. We varied the number of child nodes between 1-6 by 1. We varied subtree depth and minimum number of samples per node between 0.60-0.90 by 0.10. For each hyperparameter combination, we trained models using 1,000 iterations and an early stopping criterion of 20 iterations.

**Deep neural network.** To develop models, we utilized the *Tensorflow* [2] and *Keras* [3] R packages. We tuned the following hyperparameters using an exhaustive grid search. We varied batch size between 64, 128, and 256. We varied learning rate between 0.001, 0.01, and 0.10. We experimented with various architectures in our training data. Our best-performing architecture contained expanding and contracting dense layers based on the number of variables that passed marginal screening *p* in that training set, and a series of dropout layers with a rate of 30%. Variables were first passed through a normalization layer. See below for a table outlining this architecture. For each hyperparameter combination and architecture, we trained models using binary crossentropy loss and an adam optimization with 1,000 epochs and an early stopping criterion (patience) of 100 epochs.

**Table 1.** Best-performing deep neural network architecture.

| **Layer** | **Description** |
| --- | --- |
| 1 | Dense layer of 4*p* nodes with reLu activation function |
| 2 | Dropout layer with 30% rate |
| 3 | Dense layer of 2*p* nodes with reLu activation function |
| 4 | Dropout layer with 30% rate |
| 5 | Dense layer of *p* nodes with reLu activation function |
| 6 | Dense layer of *p*/2 nodes with reLu activation function |
| 7 | Dense layer of *p*/4 nodes with reLu activation function |
| 8 | Dense layer of 1 node with sigmoid activation function |

References

1. Chen, T., & Guestrin, C. *XGBoost: A Scalable Tree Boosting System*, in Proceedings of the 22nd ACM SIGKDD International Conference on Knowledge Discovery and Data Mining, 2016. San Francisco, California, USA. p. 785-794.
2. Abadi, M., Agarwal, A., Barham, P., Brevdo, E., Chen, Z., Citro, C., Corrado, G.S., Davis, A., et al. *TensorFlow: Large-scale machine learning on heterogeneous systems*, 2015. tensorflow.org.
3. Falbel, D., Allaire, J.J., & Chollet, F. *R interface to ‘Keras’.* keras.rstudio.com.
