# Supplementary material for: Leveraging nonlinear relationships and interactions to improve 30-day pneumonia readmission machine learning models
Source: PLoS One. 2026 Jun 5;21(6):e0349804. doi: 10.1371/journal.pone.0349804 (PMC13240904; doi:10.1371/journal.pone.0349804)
Supplement: S3 File — This file presents results from calculating SHAP values without requiring calculations for binary variables to be within patients that endorsed that binary variable. (DOCX) [file pone.0349804.s003.docx]

**S3 File. Global SHAP values**

In Fig 1, we show the top ten most influential model predictors, ranked by absolute SHAP values without the restriction that values for binary variables were calculated within patients endorsing that variable. The most significant predictors in LR models included first and last hemoglobin, hematocrit, and MCHC. The XGBoost model also highlighted the importance of first MCHC. As well, it placed importance to last BUN, body mass index, inpatient history, and fluid and electrolyte disorders. The DNN model identified first and last BUN values and last pulse as top predictors, followed by last hematocrit and emergency encounter history.

**Fig 1:** **SHAP (SHapley Additive exPlanations) values of model predictors**

**
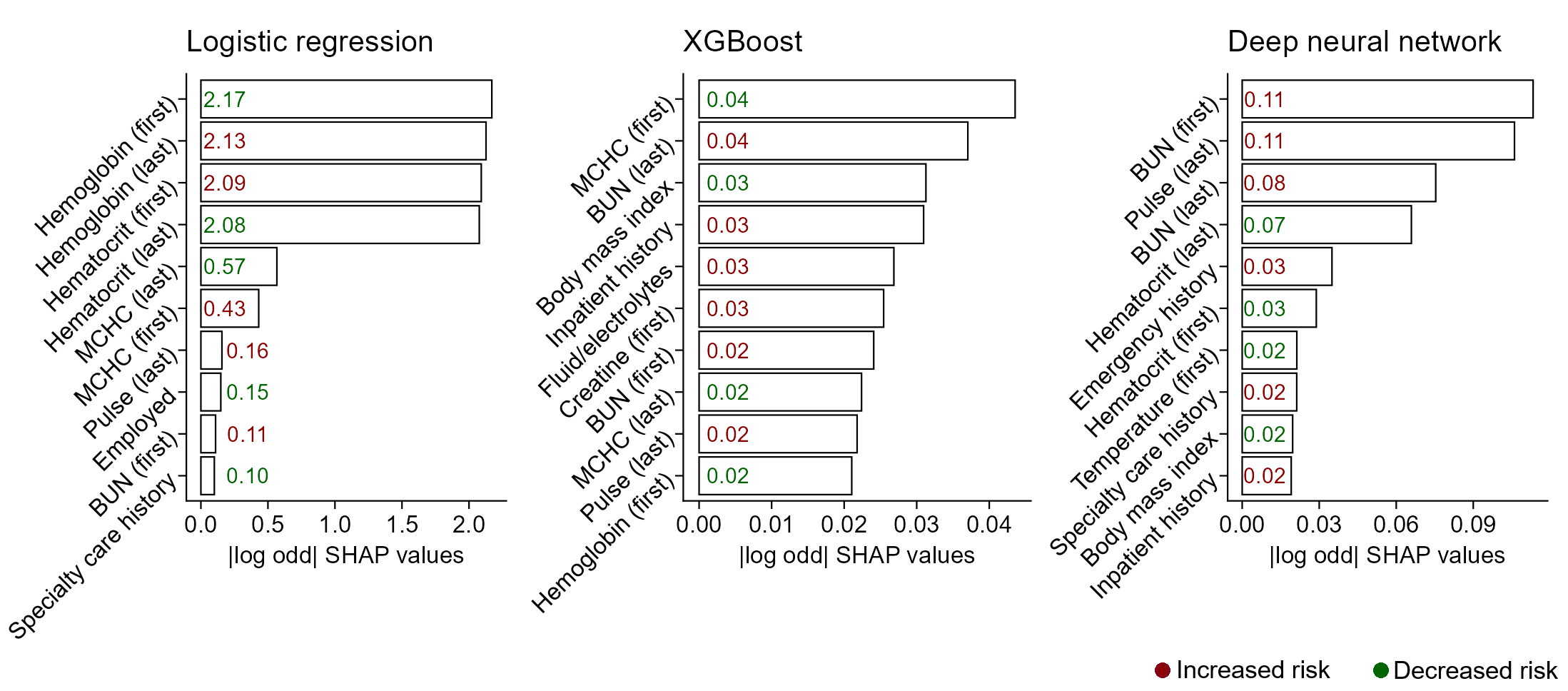
**
