# Supplementary material for: Leveraging nonlinear relationships and interactions to improve 30-day pneumonia readmission machine learning models
Source: PLoS One. 2026 Jun 5;21(6):e0349804. doi: 10.1371/journal.pone.0349804 (PMC13240904; doi:10.1371/journal.pone.0349804)
Supplement: S4 File — This file provides summarized results of a post hoc analysis where primary analyses were conducted without the initial marginal screening process. This file also includes a table of performance metrics. (DOCX) [file pone.0349804.s004.docx]

**S4 File. Running models without the marginal screening process**

In Table 1, we summarize the performance of all models. For the LR model, the average AUROC was 0.59 (95% CI: 0.52-0.65), and the AUPRC was 0.15 (0.11-0.21). The average Youden index was 0.19 (0.10-0.28). At this threshold, the average sensitivity was 0.51 (0.24-0.74), with a positive predictive value of 0.16 (0.11-0.25) and a specificity of 0.67 (0.42-0.91). An average of 9% (4-15) of patients had a readmission in the lowest decile of risk scores, while 18% (9-27) did so in the highest. Our more advanced ML models performed similarly, providing comparable average metrics and mostly overlapping confidence intervals across all models. Note, the AUROC and percentage of patients that had a readmission in the lowest decile of risk scores appeared lowest for logistic regression, although as mentioned above, confidence intervals overlapped substantially. When visually inspecting calibration plots along with Brier scores, all models performed relatively similarly. Notably, from calibration plots (data not shown), all models consistently predicted higher probabilities of readmission than actually observed across the risk spectrum.

**Table 1: Average performance of models in testing splits**

|  | **Model** | | |
| --- | --- | --- | --- |
| **Performance,**  **M (95% CI)** | **Logistic regression** | **XGBoost** | **Deep neural network** |
| **AUROC** | 0.59 (0.52-0.65) | 0.61 (0.55-0.68) | 0.63 (0.55-0.69) |
| **AUPRC** | 0.15 (0.11-0.21) | 0.15 (0.12-0.21) | 0.14 (0.06-0.22) |
| **Brier score** | 0.09 (0.09-0.10) | 0.09 (0.09, 0.09) | 0.09 (0.09-0.09) |
| **Proportion** | 0.34 (0.10-0.59) | 0.36 (0.07-0.70) | 0.37 (0.17-0.69) |
| **Youden Index** | 0.19 (0.10-0.28) | 0.22 (0.13-0.33) | 0.24 (0.14-0.33) |
| **Sensitivity** | 0.51 (0.24-0.74) | 0.56 (0.23-0.87) | 0.59 (0.37-0.89) |
| **PPV** | 0.16 (0.11-0.25) | 0.17 (0.12-0.36) | 0.16 (0.12-0.23) |
| **Specificity** | 0.67 (0.42-0.91) | 0.66 (0.32-0.95) | 0.65 (0.33-0.85) |
| **High-risk patients** |  |  |  |
| Bottom decile | 0.09 (0.04-0.15) | 0.06 (0.02-0.11) | 0.04 (0.00-0.10) |
| Top decile | 0.18 (0.09-0.27) | 0.19 (0.13-0.27) | 0.19 (0.10-0.25) |
| Notes: AUROC=area under the ROC curve. AUPRC=area under the precision-recall curve. PPV=positive predictive value. XGBoost=extreme gradient boosting model. | | | |
